# Underlying Mechanism of Antimicrobial Activity of Chitosan Microparticles and Implications for the Treatment of Infectious Diseases

**DOI:** 10.1371/journal.pone.0092723

**Published:** 2014-03-21

**Authors:** Soo Jin Jeon, Manhwan Oh, Won-Sik Yeo, Klibs N. Galvão, Kwang Cheol Jeong

**Affiliations:** 1 Emerging Pathogens Institute, University of Florida, Gainesville, Florida, United States of America; 2 Department of Animal Sciences, Institute of Food and Agricultural Sciences, University of Florida, Gainesville, Florida, United States of America; 3 Department of Large Animal Clinical Sciences, College of Veterinary Medicine,University of Florida, Gainesville, Florida, United States of America; 4 D. H. Barron Reproductive and Perinatal Biology Research Program, University of Florida, Gainesville, Florida, United States of America; University Paris South, France

## Abstract

The emergence of antibiotic resistant microorganisms is a great public health concern and has triggered an urgent need to develop alternative antibiotics. Chitosan microparticles (CM), derived from chitosan, have been shown to reduce *E. coli* O157:H7 shedding in a cattle model, indicating potential use as an alternative antimicrobial agent. However, the underlying mechanism of CM on reducing the shedding of this pathogen remains unclear. To understand the mode of action, we studied molecular mechanisms of antimicrobial activity of CM using *in vitro* and *in vivo* methods. We report that CM are an effective bactericidal agent with capability to disrupt cell membranes. Binding assays and genetic studies with an *ompA* mutant strain demonstrated that outer membrane protein OmpA of *E. coli* O157:H7 is critical for CM binding, and this binding activity is coupled with a bactericidal effect of CM. This activity was also demonstrated in an animal model using cows with uterine diseases. CM treatment effectively reduced shedding of intrauterine pathogenic *E. coli* (IUPEC) in the uterus compared to antibiotic treatment. Since Shiga-toxins encoded in the genome of bacteriophage is often overexpressed during antibiotic treatment, antibiotic therapy is generally not recommended because of high risk of hemolytic uremic syndrome. However, CM treatment did not induce bacteriophage or Shiga-toxins in *E. coli* O157:H7; suggesting that CM can be a potential candidate to treat infections caused by this pathogen. This work establishes an underlying mechanism whereby CM exert antimicrobial activity *in vitro* and *in vivo,* providing significant insight for the treatment of diseases caused by a broad spectrum of pathogens including antibiotic resistant microorganisms.

## Introduction

Chitosan has been highlighted as a potential candidate for targeting antibiotic resistant microorganisms due to a broad spectrum of antimicrobial activity and biocompatibility [1], [2], [3], [4], [5], [6]. Chitosan, a deacetylated derivative of chitin, is a linear biopolymer composed of β-(1–4)-linked N-acetyl-D-glucosamine [7]. Recently, chitosan derived from shrimp has been recognized as a Generally Recognized As Safe (GRAS) for general use in foods by the US Food and Drug Administration [8]. In addition, Japan and Korea have approved chitosan as a food additive since 1983 and 1995, respectively [9].

Various theories have been proposed to explain the mode of action leading to the antimicrobial activity of chitosan [1], [10], [11], [12]. Though the exact mechanism has yet to be elucidated, the intracellular leakage hypothesis is widely accepted [1], [10], [11], [12]. In this mechanism, positively charged chitosan binds to the negatively charged bacterial surface leading to altered membrane permeability, which results in leakage of intracellular constituents causing cell death [3], [5], [11]. However, it has been reported that antimicrobial activity of chitosan is limited to acidic conditions due to the loss of positive charges on the amino group at neutral pH [3], [5]. This restricts the use of chitosan as an antimicrobial agent at neutral pH.

Recently, we found that chitosan microparticles (CM), derived from chitosan by cross-linking, reduced pathogenic *Escherichia* coli O157:H7 shedding in cattle. This result was unexpected because the gastrointestinal (GI) tract normally maintains neutral pH where antimicrobial activity of chitosan is abolished [13]. In this earlier study, CM, administered orally with feeds, significantly shortened the duration of *E. coli* O157:H7 shedding from 13.8 days to 3.8 days and reduced the total number of this pathogen in cattle. We observed that the pathogen was completely removed from the GI tract in 60% of the calves, indicating that CM retain activity at neutral pH. These data suggest that CM can be a great candidate to intervene enteric pathogens. Although we suggested that reduction of *E. coli* O157:H7 by oral CM administration might be a result of the pathogen binding activity of CM, the previous study failed to differentiate whether the reduction of *E. coil* O157:H7 was mediated by antimicrobial activity or detaching activity of CM in the GI tract [13].

This study was designed to address the mode of action of CM by identification of binding targets in *E. coli* O157:H7. In addition to the measurement of antimicrobial activity of CM *in vitro,* an *in vivo* assessment was conducted using cows with uterine diseases to evaluate the potential for clinical application. Here, we present our findings that CM specifically interact with a bacterial surface protein, Outer Membrane Protein A (OmpA), and this interaction is coupled with antimicrobial activity. *In vivo* CM efficacy evaluated in cows with uterine diseases confirmed that CM are effective in reducing the disease-causing agent, implying potential use of this agent for disease treatment.

## Materials and Methods

### Ethics statement

Standard practices of animal care and use were applied to animals used in this project. Research protocols were approved by the University of Florida Institutional Animal Care and Use Committee (IACUC Protocol #: 201207405).

### Preparation of chitosan microparticles

CM were prepared as described previously [14]. Briefly, chitosan was purchased from Sigma-Aldrich (448869-250G, SIGMA-ALDRICH) and a 0.25% of chitosan solution (w/v) was prepared with 2% acetic acid (v/v) and 1% tween 80 (v/v). For cross-linking of chitosan, 10% of sodium sulfate (w/v) was added dropwise to the chitosan solution during stirring and sonication for 20 min. When the chitosan solution became cloudy, the chitosan microparticles were collected by centrifugation at 6000 rpm for 10 min and washed with MiliQ Water three times. The size and structure of CM were analyzed by scanning electron microscopy (EVO MA10 XVP, Carl Zeiss), and the distribution of particle size was determined by the diameter of 200 particles using image J 1.46r (National Institutes of Health, USA).

### Bacterial strains

Antimicrobial activity of CM was assessed using various bacteria. *Escherichia coli* O157:H7 EDL933 (ATCC48935), Intrauterine pathogenic *Escherichia coli*, *Salmonella enterica* CDC3041-1, and *Klebsiella pneumoniae* were grown in LB medium and tested; *Vibrio cholera* 395 classical O1, grown in LB medium supplemented with 1% NaCl, and *Streptococcus uberis*, grown in Brain Heart Infusion broth, were also tested. The wild-type strain and the mutants are listed in Table 1.

**Table 1:** Strains and plasmids.

Strains	Description	References
*E. coli* EDL933	*E. coli* O157:H7, wild-type	ATCC48935
KCJ688	EDL933 + pEYFP	This study
KCJ832	*Δeae*::kan + pEYFP	This study
KCJ846	*ΔecpA*::kan + pEYFP	This study
KCJ834	*ΔcsgA*::kan + pEYFP	This study
KCJ848	*ΔompA*::kan + pEYFP	This study
KCJ826	*ΔespA*::kan + pEYFP	This study
KCJ830	*ΔfimA*::kan + pEYFP	This study
KCJ838	*ΔfliA*::kan + pEYFP	This study
KCJ824	*ΔirgA*::kan + pEYFP	This study
KCJ844	*ΔlpfA*::kan + pEYFP	This study
KCJ828	*ΔnanA*::kan + pEYFP	This study
KCJ836	*ΔpgaA*::kan + pEYFP	This study
KCJ820	*ΔsfmA*::kan + pEYFP	This study
KCJ822	*Δtir*::kan + pEYFP	This study
KCJ840	*ΔyehD*::kan + pEYFP	This study
KCJ842	*ΔyfcV*::kan + pEYFP	This study
KCJ850	*ΔwaaL*::kan + pEYFP	This study
KCJ806	*ΔompA*	This study
KCJ1449	*ΔompA*+pOmpA	This study
KCJ852	IUPEC	Laboratory collection
KCJ855	*V. cholera*	Laboratory collection
KCJ165	*S. enterica*	CDC3041-1
KCJ1332	*K. pneumoniae*	Laboratory collection
KCJ145	*S. uberis*	Laboratory collection
pEYFP	Yellow Fluorescence Protein	Clontech
pKD4	Used as *kan* template	[15]
pKD46	Red recombinase expression plasmid	[15]

### *In vitro* antimicrobial activity assay

A single colony of each strain was inoculated in 5 ml of appropriate broth medium and incubated at 37°C with shaking at 200 rpm overnight. The next day, bacterial cultures were diluted 1:100 in fresh medium and again incubated at 37°C until reaching a late-log phase. Approximately 5×10^4^ CFU/ml of bacteria were inoculated into 2 ml of LB medium (pH 5–9) containing CM (0.05%–0.2%). After 6 h, bacteria diluted up to 10^−8^ were plated on appropriate agar and incubated at 37°C overnight to count colony-forming unit (CFU). Reduction in the number of CFU represented the antimicrobial activity.

### Live/Dead viability assay

Bacterial viability was determined using a live/dead assay (Molecular Probes, Inc., Eugene) following manufacturer's instructions. Briefly, 5×10^4^ CFU/ml of *E. coli* O157:H7 was incubated with 0.2% CM at 37°C for 3 h and then incubated at room temperature in the dark with SYTO 9 and propidium iodide. After a 15 min incubation, bacteria were observed using the fluorescence microscope (Leica Microsystems Wetzlar GmbH, Germany). Viability was determined by fluorescence colors that bacteria emit (i.e. dead bacteria present red fluorescence, and live bacteria present green fluorescence). As controls, bacteria were treated with 70% isopropyl alcohol for dead bacteria or untreated for live bacteria.

### Gene deletion and complementation

Genes of interest were deleted in the chromosomal DNA of *E. coli* O157:H7 by the PCR one-step λ Red recombinase method [15]. Briefly, the kanamycin resistance gene was amplified using a template plasmid pKD4 and primers with 50-nt extensions that are homologous to regions adjacent to a targeted gene (Table 2). The PCR product was electrophorated into the *E. coli* O157:H7 with the Red system expression plasmid pKD46. The electrophorated *E. coli* O157:H7 was added to 1 ml of LB, incubated at 37°C for 1 h, and then plated onto LB agar with kanamycin for selection.

**Table 2:** Oligonucleotides.

Names	Genes	Sequences
KCP021	ompA-F^m^	ATAAGTACCGCATAAAACCTACTATTGCTCCGCGTTTTTTACTGTAGGCTGGAGCTGCTTCG
KCP022	ompA-R^m^	GGGGCGTCGTCGCCCCAAAAAGATGGTCTGCTCTTGAATTCGCATATGAATATCCTCCTTAGTTCC
KCP023	ecpA-F^m^	CCCTGTAGTGCAGGAGTTAAGTTGAGCCCTTCTTTATGTTACTGTAGGCTGGAGCTGCTTCG
KCP024	ecpA-R^m^	TCGACCCCCCGATGGGGACGACCATGTAGTCTCTCTAATTGACATATGAATATCCTCCTTAGTTCC
KCP025	fimA-F^m^	CATGTCGATTTAGAAATAGTTTTTTGAAAGGAAAGCAGCATGTGTAGGCTGGAGCTGCTTCG
KCP026	fimA-R^m^	GTCGCATCCGCATTAGCAGCACCCGGGGTTGCCTCGCCCATATGAATATCCTCCTTAGTTCC
KCP027	pgaA-F^m^	ATGTCTCTCTCTAAAACCGTTATGTACCTCATTATGTCCTACTGTAGGCTGGAGCTGCTTCG
KCP028	pgaA-R^m^	CCTCTATAAATAAAGGTAATGCATTGTATAAATAGGAATTTTCATATGAATATCCTCCTTAGTTCC
KCP029	nanA-F^m^	TAGTCTGTTCGTAGTGAAGTCTCCATAAATACCGTTGCTTAATGTAGGCTGGAGCTGCTTCG
KCP030	nanA-R^m^	GGGGAGTGGGCCATCCCCGCTCGCTCCCCTTTGTTGAGTGGGCATATGAATATCCTCCTTAGTTCC
KCP031	csgA-F^m^	TTCCATTCGACTTTTAAATCAATCCGATGGGGGTTTTACATGTGTAGGCTGGAGCTGCTTCG
KCP032	csgA-R^m^	GGGCTTGCGCCCTGTTTCTTTAATACAGATGATGTATTAGTACATATGAATATCCTCCTTAGTTCC
KCP063	lpfA-F^m^	TAAAATTATGTAGTTCTAAAAGAAAAATTACATTAAAAAATTTGTAGGCTGGAGCTGCTTCG
KCP064	lpfA-R^m^	CTGATTACCGCCGTTAATGCGGCGGTAAACATTTTGCCTGCTCATATGAATATCCTCCTTAGTTCC
KCP065	eae-F^m^	ATTAAATAAAGAGTAAGATTGAGTAACACCACCTCGGTATTGTGTAGGCTGGAGCTGCTTCG
KCP066	eae-R^m^	GGCTGATTTTGTTATGTATAAAATCGGCCCCACCAATACCTTCATATGAATATCCTCCTTAGTTCC
KCP067	tir-F^m^	TTCCTAACAATAGATAAATGTATTTTATTTTTCCTCTATAAATGTAGGCTGGAGCTGCTTCG
KCP068	tir-R^m^	AAGGGGGGAGGGAGGGAGATTTATTTTACTAATACCTATATACATATGAATATCCTCCTTAGTTCC
KCP069	espA-F^m^	TAAAAAAACAAAAGGACTCTTTTTAATAGTTCTCCATATATCTGTAGGCTGGAGCTGCTTCG
KCP070	espA-R^m^	TCTGTCCCATAGCAATAAATGCAATTCGTATCAATAGAGGCCCATATGAATATCCTCCTTAGTTCC
KCP071	fliA-F^m^	CTATTAGTACGGCTATTGAGTATATTGCGTCCCGACAAATAGTGTAGGCTGGAGCTGCTTCG
KCP072	fliA-R^m^	CTTGAGGACCATCAGTTTCAATTTCACGCCGTAAATGACTGCCATATGAATATCCTCCTTAGTTCC
KCP073	yehD-F^m^	TCTGAGAATTGTTTTGTTTTATTTGAATAATTCCTTACGTAGTGTAGGCTGGAGCTGCTTCG
KCP074	yehD-R^m^	GTAAAATTGAATGACTTTTTTGTTCTACTAATAAAATTTATACATATGAATATCCTCCTTAGTTCC
KCP075	yraH-F^m^	ATTAAATATGATTATGTACTTGTTACAAGGATAAGGTTATAATGTAGGCTGGAGCTGCTTCG
KCP076	yraH-R^m^	GAAAAGGGCGTTATCTGAAAGGTCAGATAACGCCGTAACGTACATATGAATATCCTCCTTAGTTCC
KCP077	sfmA-F^m^	TACTATCAGTGTCTTAAATAAAGTAATCGGTTATATACGGATTGTAGGCTGGAGCTGCTTCG
KCP078	sfmA-R^m^	GTTATAAGACGTGATATATAATTCAAAACAACGTGGTTTTGACATATGAATATCCTCCTTAGTTCC
KCP079	yfcV-F^m^	GTTACAAATATAAAAATTAATAAATACCAAATTCCTGTTTATTGTAGGCTGGAGCTGCTTCG
KCP080	yfcV-R^m^	GGTAAATTTTTGGGGTATCGGGCTTTCGATACCCCCATAGTGCATATGAATATCCTCCTTAGTTCC
KCP708	ompA-F	ATGATAACGAGTCGACAAAAATGAAAAAGACAGCTATCGCGATTG
KCP709	ompA-R	CCCGGAATTCTTAAGCTTGCGGCTGAGTTACAAC

m Primers were designed for gene inactivation in chromosomal DNA of *E. coli* O157:H7.

A *ΔompA* strain was transformed with a pUC18 carrying the *ompA* gene to create a complement strain (*ΔompA*+pOmpA) using primers PKC708 and PKC709 (Table 2). We confirmed the absence of the *ompA* gene in the *ΔompA* mutant and the restoration of *ompA* in the *ΔompA*+pOmpA strain by PCR. A growth curve was measured (OD_600_) every 30 min for 12 h using a plate reader (Gen5, BioTek). The complementation was measured by an *in vitro* binding assay and an antibacterial activity assay. These strains were stained with the blue-fluorescent DAPI (Invitrogen) and observed by fluorescence microscopy (Leica Microsystems Wetzlar GmbH, Germany).

### *In vitro* CM binding assay

*E. coli* O157:H7 was loaded on 6-mm wells of CM-coated, poly-Lysine-coated, or uncoated slides to examine cell attachment to CM (Electron Microscopy Sciences of Hatfield, PA). To prevent desiccation of bacteria, the glass was kept in a humid chamber during incubation. After incubation, the glass was washed 3 times with phosphate buffered saline (PBS) to remove unbound cells and added with antifade reagent (ProLong Gold, Invitrogen) to protect fluorescent dyes from photobleaching. The mounted wells were observed by fluorescence microscopy (Leica Microsystems Wetzlar GmbH, Germany). To identify the molecular target of CM in the cell attachment, *E. coli* O157:H7 wild-type and 15 mutants carrying pEYFP (Clontech, Table 1) were grown in LB medium at 37°C for 3 h with isopropyl-β-D-thiogalactopyranoside (IPTG). These strains in late log phase were harvested at 4000 rpm for 1 min. Approximately 10^6^ CFU/ml of each strain prepared in LB medium was incubated on a CM-coated glass. The CM-coated glass was prepared earlier by loading 20 μl of 0.1% CM on 6-mm wells of the glass. The following procedures for washing, mounting, and examination were the same as described above.

### Phage induction analysis

Bacterial cell lysis was measured by spectrophotometer to monitor phage induction. *E. coli* O157:H7 was grown in LB medium until exponential phase (OD_600_  = 0.7). Different concentrations of CM, mitomycin C, and medium alone (as a control) were added in eight replicates. Optical density of cultures was measured by spectrophotometer (Gen5, BioTek) every 30 min during 7 h with moderate shaking. Phage induction was also evaluated by sodium dodecyl sulfate polyacrylamide gel electrophoresis (SDS-PAGE). *E. coli* O157:H7 was grown in LB medium until exponential phase (OD_600_  = 0.7). CM (0.2%), mitomycin C (1 μg/ml, Fisher Scientific BP-2531-2), and medium alone were added in 10 ml of bacterial culture, respectively, followed by incubation for 18 h. To remove unlysed cells and debris, the bacterial culture was centrifuged at 4,000 rpm for 20 min at 4°C. The The supernatants were filtered through 0.22 μm-pore-size membrane filter (Fisher Scientific 09-719A) and phage particles expected to be induced by treatments were precipitated by the addition of 20% polyethylene glycol 8000 (PEG, Fluka 81268) and 2.5 M NaCl. The precipitated phage particles were incubated on ice for 30 min and then centrifuged at 14,000 rpm for 20 min. Centrifugation was repeated twice to remove all of the PEG solution. The pellets were resuspended in STE buffer (1 M Tris [pH 8], 0.5 M EDTA [pH 8], and 5 M NaCl), mixed with 5× SDS loading buffer, boiled for 5 min, and applied to an 12% polyacrylamide gel. The protein bands were visualized by staining with Coomassie blue.

### Western blot analysis of Shiga-toxin II expression

*E. coli* O157:H7 was grown in LB medium until exponential phase (OD_600_  = 0.7). CM (0.2%), mitomycin C (1 μg/ml), and medium alone were added in 10 ml of bacterial culture, respectively. The cultures of *E. coli* O157:H7 were incubated for 18 h, centrifuged at 4,000 rpm for 20 min at 4°C, and filtered through 0.22 μm-pore-size membrane filter to remove bacteria. For precipitation of proteins present in the supernatants, filtered cultures were mixed with 100% trichloroacetic acid (Fisher Scientific BP555-1) to create the final 20%. The mixtures were incubated on ice for 30 min and centrifuged at 14,000 rpm at 4°C for 30 min. The supernatant was carefully removed and the pellet was washed with cold acetone. The cold acetone needed to be aspirated following centrifugation at 14,000 rpm at 4°C for 15 min. The remaining pellets were suspended with sterile water and protein concentrations were measured by spectrophotometer (Nanodrop 1000, Thermo Scientific). Subsequently, the proteins were separated by 12% sodium dodecyl sulfate polyacrylamide gel and transferred to a polyvinylidene difluoride membrane (0.45 μm pores, Immobilon-P, Millipore) for western blot. The membrane was blocked in 5% skim milk in TBST (10 mM Tris-HCL [pH 7.4], 150 mM NaCl, and 0.05% Tween-20) for 1 h at RT and incubated with monoclonal Verotoxin II-α subunit antibody (Meridian, Life Science Inc.) overnight at 4°C. After washing with TBST, the membrane was incubated at RT for 45 min with HRP-conjugated secondary antibody (GE Healthcare) diluted 1:10000 in TBST. After washing with TBST, the membrane was incubated with a chemiluminescent substrate (ECL Plus, GE Healthcare), and exposed to Kodak BioMax film (Carestream Kodak X-Omat LS film, F1274 Sigma).

### Animal management

Cows used in this experiment were housed in freestall barns equipped with fans and sprinklers that were activated when ambient temperature exceeded 18°C. Barns were cleaned twice daily and freestalls were bedded twice a week with sand. Cows were fed totally mixed ration twice daily formulated to meet or exceed the nutrient requirements of a lactation Holstein cow weighing 650 Kg of body weight and producing 45 Kg of 3.5% fat corrected milk per day. Fresh water was available ad libitum. Cows were vaccinated and treated for common diseases according to standard operating procedures (SOP) developed with participation of the veterinarians from the University of Florida, college of veterinary medicine, food animal reproduction and medicine service.

### *In vivo* antimicrobial activity of CM in cows with uterine diseases

For *in vivo* animal experiments, Holstein cows (8–10/group) having metritis (inflammation of all layers of the uterus) were used for this study. Metritis was diagnosed by the presence of red-brownish fetid uterine discharge at either 4, 7, or 10 days postpartum. Cows (n = 18) were randomly assigned to one of 2 treatments as they were diagnosed with metritis: CM treatment, Cows (n = 8) were treated for 5 days with 8 g of CM dissolved in 10 ml of sterile water via intrauterine infusion; Ceftiofur treatment, cows (n = 10) were intramuscularly treated for 5 days with 2.2 mg/kg of Ceftiofur hydrochloride (Excenel, Zoetis, Zoetis Inc., Madison, NJ). The uterus of a cow with metritis has a volume of approximately 4 L; therefore, the dose of CM used was approximately 0.2%. For enumeration of *E. coli* in uterus, uterine swabs were daily collected for 7 days starting at day 0. Swab samples were suspended in 0.1% (w/v) peptone and serially diluted, and plated on CHROMagar (CHROMagar, Paris, France) in duplicates. Plates were incubated at 37°C overnight and the numbers of CFU/ml were counted.

### Statistical analysis

All experiments were carried out in triplicate, and average values with standard error of the mean are reported. Data were compared with student's t test using the GraphPad Prism InStat 3.1. *P* values less than 0.05 were considered as statistically significant.

## Results

### Antimicrobial properties of CM

**Figure 1:**
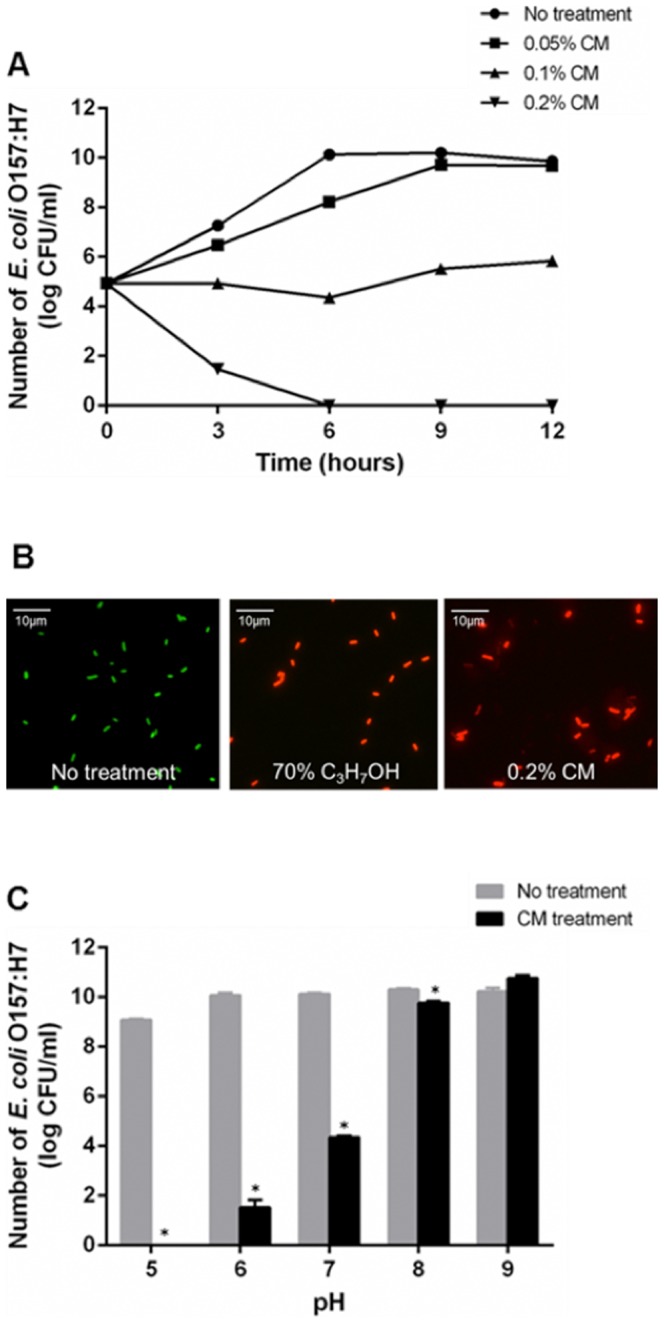
Antimicrobial activity of chitosan microparticles. (A) Survival curve of *E. coli* O157:H7 during CM treatment. *E. coli* O157:H7 was grown with CM at 0% (circle), 0.05% (square), 0.1% (triangle), or 0.2% (inverted triangle), and each time point represents the mean values and standard error of means (SEM) of colony forming units (CFU) recovered from triplicate test tubes. (B) LIVE/DEAD viability assay. *E. coli* O157:H7 was incubated with 0.2% CM at 37°C for 3 h and then incubated with SYTO 9 (green, live) and propidium iodide (red, dead) for 15 minutes, and then bacteria were observed using the fluorescence microscope (Leica Microsystems Wetzlar GmbH, Germany). Fluorescent micrograph of *E. coli* O157:H7 treated with either 0% CM (left), 70% isopropanol (middle), or 0.2% CM. Results shown are representative of three independent experiments. (C) pH effect on antimicrobial activity of CM. *E. coli* O157:H7 was incubated for 6 h in the presence of 0.1% CM in LB broth at various pH (ranging from pH 5 to pH 9) and viable cells were counted using direct plating method; Mean values ± SEM are plotted from three independent experiments; * *P*<0.05, *t*-test.

CM have been shown to reduce *E. coli* O157:H7 from in cattle when administered orally [13], but the mode of action was not clearly understood. To understand if the *E. coli* O157:H7 reduction was caused by either scrubbing or antimicrobial action, antimicrobial activity was determined by a standard plating method after incubation of *E. coli* O157:H7 with CM at various concentrations, ranging from 0% to 0.2%. CM showed a concentration dependent bactericidal activity against *E. coli* O157:H7 (Fig. 1A). Of all concentrations examined, 0.2% CM showed the most antimicrobial activity resulting in complete inhibition of *E. coli* O157:H7 during 6 h of incubation. Growth of *E. coli* O157:H7 was inhibited by 0.1% CM, showing steady level of *E. coli* O157:H7 numbers during 12 h of incubation. Although bacterial growth reached the level in controls (no CM treatment), 0.05% CM treatment showed decreased growth rate until 9 h post incubation.

Using the Live/Dead assay, live bacteria with intact membranes emit green fluorescence (stained with SYTO 9); whereas, bacteria with damaged membranes emit red fluorescence (stained with propidium iodide) due to the penetration of this dye into the cytosol through the damaged membranes. As shown in Fig 1B, dead *E. coli* O157:H7 cells, (permeabilized with 70% isopropanol as a positive control) emitted red fluorescence; whereas, live intact cells (no treatment) emitted green fluorescence. *E. coli* O157:H7 treated with 0.2% CM was stained in red fluorescence, indicating that the bacterial membranes were permeabilized by CM treatment. These data demonstrate the bactericidal activity of CM.

Previous studies have shown that antimicrobial activity of chitosan is abolished at neutral pH due to the pKa value of amino group in chitosan (pKa = 6.5) [11]. However, CM feeding to cows revealed a reduction of *E. coli* O157:H7 numbers in cattle [13], suggesting some antimicrobial activity at neutral pH. This led us to test antimicrobial activity at different pH, including the neutral pH. Although the strongest antimicrobial activity was observed at acidic pH (pH5), CM still had significant antimicrobial activity at pH7 (Fig. 1C). These data suggest that the bactericidal activity was likely the reason for the reduction of *E. coli* O157:H7 shedding in a previous study [13]. This unexpected antimicrobial activity of CM at pH7, where positively charged amino groups are unlikely, led us to speculate that CM may exert antimicrobial activity at neutral pH, which is distinct to the proposed charge dependent activity at acidic pH.

### Identification of CM binding target in *E. coli* O157:H7

**Figure 2:**
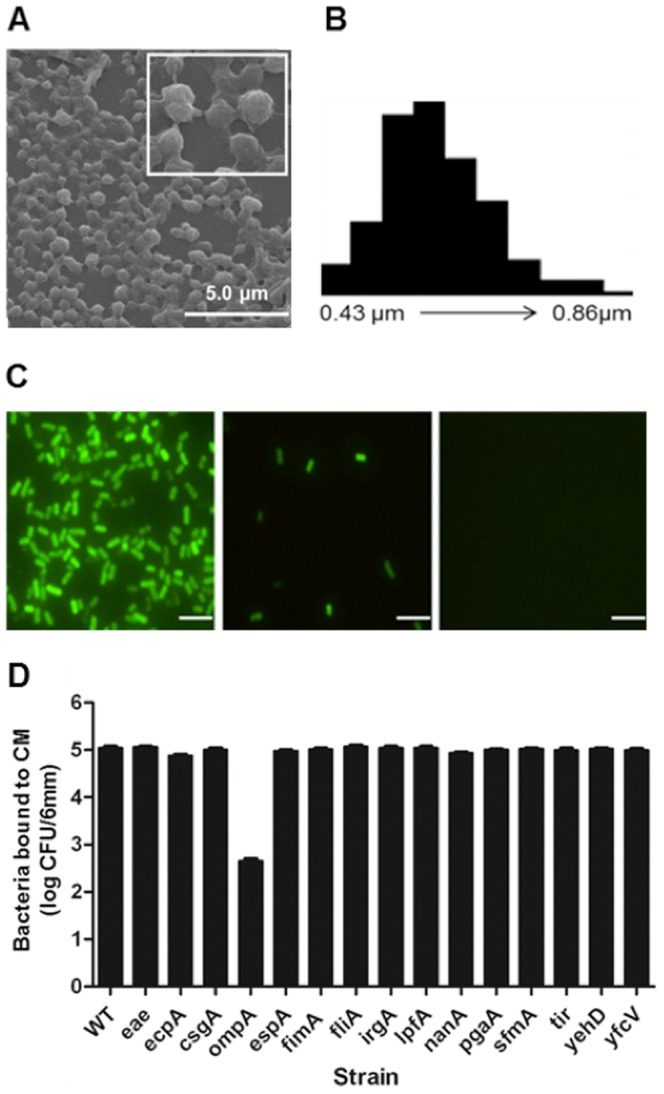
Identification of CM binding target using *in vitro* CM binding assay. (A) Scanning electron microscopy micrograph of CM. Inset is magnified image of CM. (B) The distribution of particle size was determined by measuring the diameter of 200 particles using image J software. (C) *In vitro* binding assay of *E. coli* O157:H7. Bacteria cells carrying pEYFP grown in the presence of IPTG were harvested and incubated on CM-coated (left), poly-lysine-coated (middle), or no-coated slides (right). Bacteria in 6-mm wells were observed by fluorescence microscopy. Scale bars represent 5 μm. (D) Identification of CM binding target. *E. coli* O157:H7 strains were grown in broth and cells were collected to incubate on CM-coated slides. Bound cells were enumerated by fluorescence microscopy. Strains used were: wild type plus vector (KCJ688), *Δeae* plus vector (KCJ832), *ΔecpA* plus vector (KCJ846), *ΔcsgA* plus vector (KCJ834), *ΔompA* plus vector (KCJ848), *ΔespA* plus vector (KCJ826), *ΔfimA* plus vector (KCJ830), *ΔfliA* plus vector (KCJ838), *ΔirgA* plus vector (KCJ824), *ΔlpfA* plus vector (KCJ844), *ΔnanA* plus vector (KCJ828), *ΔpgaA* plus vector (KCJ836), *ΔsfmA* plus vector (KCJ820), *Δtir* plus vector (KCJ822), *ΔyehD* plus vector (KCJ840), and *ΔyfcV* plus vector (KCJ842).

To test if CM binding to *E. coli* O157:H7 is necessary for antimicrobial activity, we used *in vitro* binding assay of CM. CM were generated by ion gelation with sodium sulfate [14], [16], and we visualized and the measured particles using scanning electron microscopy analysis. The diameter of the prepared CM was 0.6±0.076 μm (mean ± SD, n = 200) with spherical shape and rough surface (inset) structure (Fig. 2A and 2B). Binding activity of CM to *E. coli* O157:H7 was measured using CM-coated glass slides (Fig. 2C). *E. coli* O157:H7 strain expressing enhanced yellow fluorescence protein (EYFP) attached to the CM and poly-lysine (positive control) coated wells, while no cells attached to the untreated wells (Fig. 2C), indicating that CM have significant binding capacity to *E. coli* O157:H7. These data suggest that a direct contact between CM and this pathogen may result in bactericidal effect. From these findings, we hypothesized that surface exposed molecules in *E. coli* O157:H7 might bind to CM. To determine if the observed binding was related to a specific bacterial gene, we constructed mutant strains lacking individual gene in *E. coli* O157:H7 using λ-Red homologous recombination [15]. Total of 15 genes, which are known to be important for bacterial adhesion during colonization, were selected to identify potential binding partners of CM. The target genes are summarized in Table 1. The constructed mutant strains were used for *in vitro* CM binding assay to identify potential binding partners. A total of 10^4^ cells were loaded into the CM-coated wells, incubated, and then unattached cells were removed by washing before enumeration with a fluorescent microscope. As shown in figure 2D, the *ΔompA* mutant shows significantly reduced binding to CM compared to the wild-type strain and other mutant strains, suggesting OmpA is a target for CM binding.

### Both OmpA and LPS are CM binding targets

**Figure 3:**
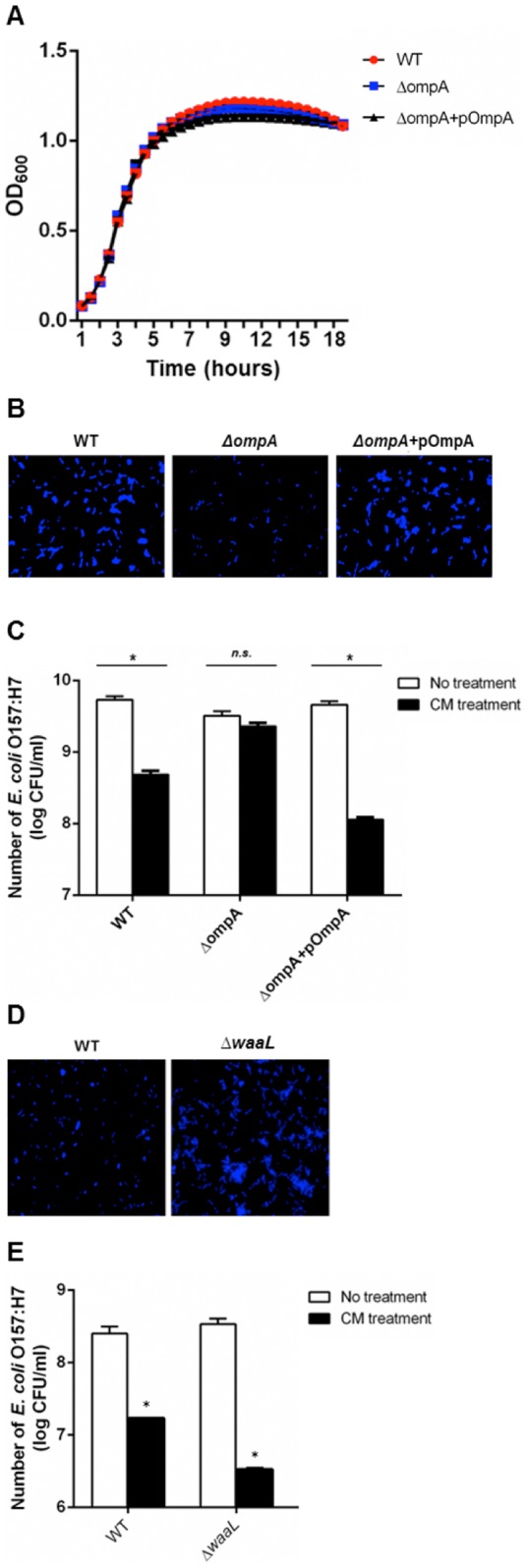
CM interaction with *E. coli* O157:H7 via OmpA and LPS is linked to antimicrobial activity. (A) Growth curve of wild-type (circle), *ΔompA* (KCJ806), and *ΔompA*+pOmpA (KCJ1449). (B) *In vitro* CM binding assay of the *ΔompA* strain. pOmpA complements the loss of binding activity of the *ΔompA* strain. Bound cells were stained with DAPI and enumerated with fluorescence microscopy. (C) Binding of CM to OmpA is linked to the antimicrobial activity of CM. Antibacterial activity of CM was attenuated in *ΔompA,* but restored with pOmpA. Strains used for antimicrobial assay were: wild-type (EDL933), *ΔompA* (KCJ806), and *ΔompA*+pOmpA (KCJ1149). (D) *In vitro* binding assay of wild-type (left) and *ΔwaaL* (right). Bound cells were stained with DAPI and enumerated with fluorescence microscopy. (E) Antimicrobial activity of CM increased in the *ΔwaaL* mutant. Strains used for antimicrobial assay were: wild-type (EDL933) and *ΔwaaL* (KCJ850). Mean values ± SEM are plotted from three independent experiments; * *P*<0.05, *t*-test.

The strains were tested to determine if the reduced CM binding activity of the *ompA* mutant was not caused by growth defect of the mutant strain. The *ΔompA* did not show any growth defect (Fig. 3A), indicating that deletion of this gene was not pleiotropic. Therefore, the reduction of CM binding (Fig. 2D) was solely caused by the *ΔompA* deletion. Functional complementation of the *ΔompA* mutant was evaluated with the CM binding assay. After CM binding and washing, the attached cells were stained with DAPI to visualize bacteria (Fig. 3B). A reduced number of the *ΔompA* mutant was observed but numbers were restored to the wild-type level by complementation (*ΔompA*+pOmpA). Taken together, these results indicate that OmpA protein specifically interacts with CM resulting in binding on the CM-coated slide.

It was noted that the *ΔompA* strain still had binding capacity to CM, suggesting that OmpA is not the only one interacting protein, but other molecules are probably involved. To study this hypothesis, we tested if lipopolysaccharide (LPS) can be a binding target based on the previous finding that purified LPS bound to chitosan *in vitro* [17]. We generated a *ΔwaaL* mutant to remove O side chain in LPS. This mutant strain has more negative charges on cell surface compared to the wild-type strains because negatively charged lipid A and core components of LPS are exposed by the deletion of O side chain [18]. Therefore, we expected to increase the CM binding activity with the mutant strain. As shown in figure 3D, an increased number of *ΔwaaL* mutant were observed on CM-coated slides compared to the wild-type strain, suggesting that LPS can bind to CM.

### Association of CM binding with antimicrobial activity

To determine if CM binding to *E. coli* O157:H7 was coupled with antimicrobial activity, it was measured in the *ΔompA* and *ΔwaaL* strains. The wild-type, *ΔompA*, and the *ΔompA* complement stains were grown to exponential phase and then incubated with CM (0.05%) for 6 h before enumeration. The growth of the wild-type and complemented strains was significantly inhibited compared to control cells; whereas, the *ΔompA* strain was not affected by CM, indicating that OmpA was necessary for antimicrobial activity (Fig. 3C). However, the *ΔompA* strain was sensitive at greater concentration at 0.1 and 0.2% (data not shown). In addition, the antimicrobial activity of CM was increased in the *ΔwaaL* strain compared to the wild-type strain (Fig. 3E), suggesting that the enhanced binding activity in the strain (Fig. 3D) increased antimicrobial activity. Together, these data indicate that the bactericidal activity of CM is coupled with the binding activity to OmpA and LPS in *E. coli* O157:H7.

### A broad-spectrum of antimicrobial activity

**Figure 4:**
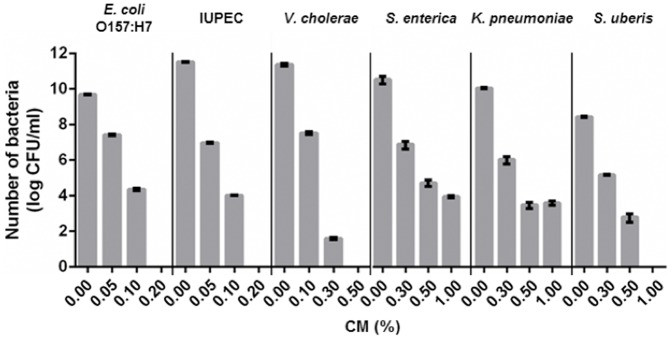
A broad antimicrobial activity of chitosan microparticles. Various strains including *E. coli* O157:H7, Intrauterine pathogenic *E. coli* (IUPEC), *V. cholerae, S. enterica, K. pneumoniae, S. uberis* were treated with CM at different concentrations and viable cells were measured after 6 h treatment; Mean values ± SEM are plotted from three independent experiments.

Antimicrobial activity of CM was tested using six important pathogens that cause disease in humans and animals. CM showed antimicrobial activity against all six pathogens with different efficacy depending on the pathogens (Fig. 4). Growth of *E. coli* O157:H7 and intrauterine pathogenic *E. coli* (IUPEC) were inhibited at 0.05%, and inactivated at 0.2%. In comparison to these *E. coli* species, *V. cholerae*, *S. enterica, K. pneumoniae*, and *S. uberis* were less sensitive to CM at 0.1 or 0.3%. *V. cholerae* was inactivated at 0.5%; whereas, *S. ubreis* was inactivated at 1%. Taken together, CM have a broad-spectrum of antimicrobial activity against important pathogens.

### *In vivo* antimicrobial activity of CM

**Figure 5:**
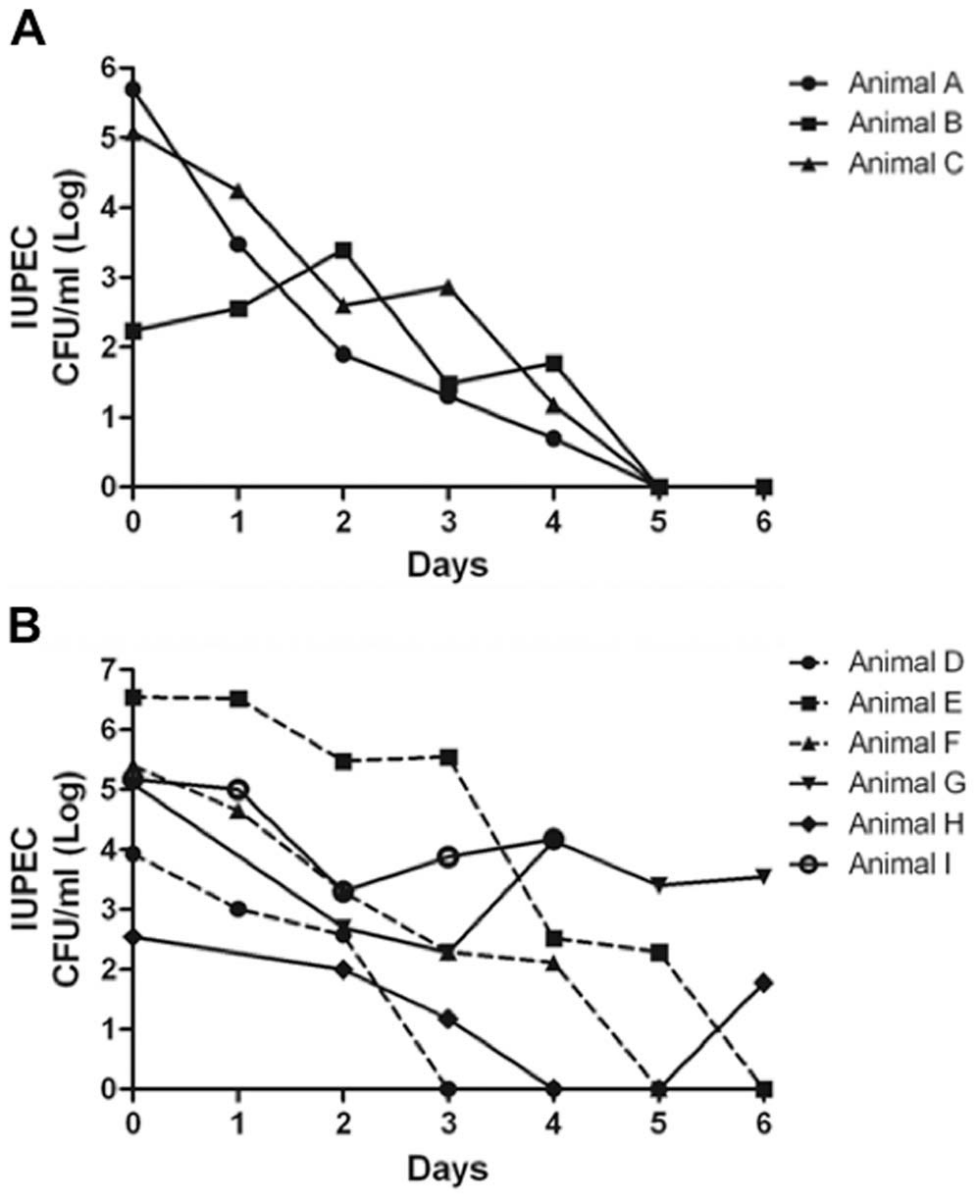
*In vivo* antimicrobial activity of chitosan microparticles. CM reduces number of intrauterine pathogenic E. coli (IUPEC) in the uterus. Dairy cattle with uterine disease were treated with either 0.2% CM (A), n = 8, via intrauterine fusion or antibiotic Ceftiofur hydrochloride (B), n = 10, systemically for five days. Swabs were collected from uterus for a seven days and IUPEC was enumerated on *E. coli* selective media CHROMagar. Plates were incubated at 37°C overnight, and the numbers of CFU/ml were counted. Numbers of IUPEC in the uterus are presented from individual animals shed this pathogen on Day 0. Solid lines represent animals without IUPEC after treatment; dashed lines represent animals with IUPEC after treatment.

We evaluated *in vivo* antimicrobial activity of CM using cows with uterine diseases as a model animal. Uterine inflammatory diseases, such as metritis and endometritis, are highly prevalent in postpartum cows [19]. The cow uterus maintains its pH near neutral (pH 6.84–7.51) [20], thus it is an ideal *in vivo* model to test CM antimicrobial activity at neutral pH. Holstein cows with uterine diseases were administered with one of two treatments: one with ceftiofur (n = 10 cows), as positive reference, which is used normally to treat uterine infections and the other with CM (n = 8 cows). The number of IUPEC from uterine swab samples collected from the treated animals were enumerated by direct plating on CHROMagar *E. coli* (CHROMagar, Paris, France). *In vivo* antimicrobial activity was measured using cows actively shedding IUPEC prior to CM or ceftiofur treatment (day 0). Three and six cows were positive for IUPEC on day 0 in CM and ceftiofur treated cows, respectively (Fig. 5). Numbers of IUPEC were monitored in uterus for seven days. IUPEC numbers recovered from CM treated cows were significantly reduced within five days in all treated animals (Fig. 5A). However, three out of five cows treated with ceftiofur still shed IUPEC on day five (Fig. 5B). Thus *in vivo* antimicrobial activity by CM was demonstrated, providing a great promise to treat infections as an alternative antimicrobial agent.

### Effect of CM treatment on phage induction and Shiga-toxin production

We further investigated the possibility if CM can be used to treat *E. coli* O157:H7 infection. Shiga-toxin is an important virulence factor in *E. coli* O157:H7 and causes deadly hemolytic uremic syndrome in humans [21], [22]. *stx* genes encoding Shiga-toxin are located in the prophage (BP-933W) in the *E. coli* O157:H7 EDL933 strain and induced when phage is triggered into the lytic cycle by antibiotic treatment [23]. Thus, antibiotic treatment is generally not recommended for patients infected with this pathogen [24].

**Figure 6:**
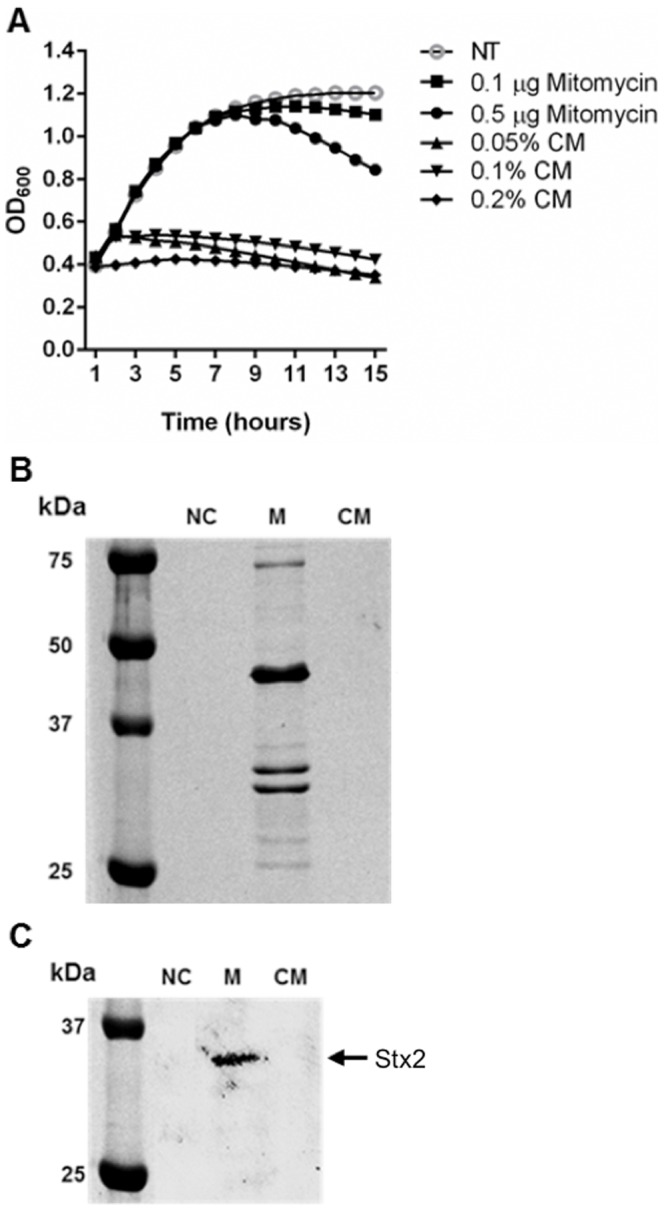
Chitosan microparticles do not induce bacterial phage and Shiga-toxin expression. (A) Phage induction was measured by a reduction of the OD_600_ of the bacterial culture with mitomycin C or CM at different concentrations. (B) SDS-PAGE analysis of phage proteins. Phage particles were obtained by PEG precipitation of cell lysate from mitomicin C-treated and CM-treated cultures. Phage proteins are visualized by Coomassie blue staining. (C) Shiga-toxin expression by CM treatment. *E. coli* O157:H7 strain was grown with CM and cell lysates were collected. Shiga-toxin expression was analyzed by Western blotting using antibody specific to Shiga-toxin. NC (negative control; without treatment), M (mitomycin C, 1 μg/ml), CM (chitosan microparticles, 0.2%).

To examine if CM have potential to treat patients infected with *E. coli* O157:H7, phage induction and Shiga-toxin production were monitored in CM treated cells. CM did not induce prophage or Shiga-toxin production (Fig. 6). Prophage induction was assessed by OD_600_ in the presence of CM at 0.05%, 0.1%, and 0.2%, along with a positive control (mitomycin C) and negative control (without treatment). Cell lysis was achieved with mitomycin C treatment (0.5 μg/ml), showing phage induction by antibiotic treatment (Fig. 6A). In the CM treated cells, cell lysis was not observed due to lack of cell growth with CM. Since it was unclear whether CM induced the phage in *E. coli* O157:H7, we detected phage particles as described previously [25]. Phage particles were collected and identified from the supernatants after CM or mitomycin C treatment. A protein profile associated with BP-933W induction was detected in supernatants from mitomycin C-treated cells as previously reported [25] but not in the supernatants of CM-treated and untreated cells (Fig 6B), indicating that CM kill bacteria prior to the phage induction. Western blot analysis for Shiga-toxin in the supernatant collected from CM-treated or mitomycin C-treated cells were conducted. Shiga-toxin was only detected in the mitomycin C-treated cells, but not in the CM-treated and untreated cells. Thus, CM exert antimicrobial activity before phage induction and Shiga-toxin production, implying CM may have potential in the treatment of infected humans.

## Discussion

Our findings hold great promise of CM as an alternative antimicrobial agent for the treatment of bacterial infections, including antimicrobial resistant microorganisms. In this paper, we have shown the underlying mechanism of antimicrobial activity of CM, which disrupts bacterial cell membranes by interactions with the outer membrane protein OmpA at neutral pH, leading to cell death. Antimicrobial activity of CM was also confirmed using an animal model with uterine disease. Furthermore, CM did not induce phage or Shiga-toxin production in *E. coli* O157:H7.

Although the antimicrobial activity of chitosan has not been clearly understood, the hypothesis that ionic interactions between positively charged amino group in chitosan and negatively charged bacterial surface molecules such as LPS in acidic conditions [17] resulting in alteration of membrane permeability, has been widely accepted [12], [26]. Based on the proposed antimicrobial mechanisms of chitosan, we assumed that CM might have the same antimicrobial mechanism as that of chitosan through the ionic interaction. However, we found that the antimicrobial activity of CM was, in part, mediated through OmpA (Fig. 3C). OmpA is an integral bacterial outer membrane protein embedded as a β-barrel structure and it contributes to the structural integrity of the bacterial cell surface [27]. In addition, it contains four surface exposed loops [28] which are involved in the recognition of many ligands including small molecules such as iron-siderophore complexes or sugars [29], [30]. Thus, we thought that the loops might be involved in the interaction with CM via ionic interaction. However, the surface-exposed loops of OmpA have a net negative charge of +1 [31], indicating that the ionic interaction between CM and the loops is unlikely.

We speculate that the CM-OmpA interaction is direct via hydrogen bond interaction. Multiple lines of evidence support our hypothesis. It has been shown that *E. coli* K1, which causes meningitis, especially in new-born babies [32], invades membranes via an interaction of OmpA with a D-glucosamine (monomer of chitosan) [33], [34]. Recently, residues critical for the hydrogen bond interactions between OmpA with D-glucosamine were identified in the loops of OmpA by a computational simulation analysis [35]. In addition, N-acetylated D-glucosamine is a critical receptor for colonization of *V. cholea* in the GI tract. *V. cholera* secretes the GlcNAc-binding protein A (GbpA) to bind N-acetylated D-glucosamine [36]. The *gbpA* mutant strain failed to attach to HT-29 epithelial cells and to colonize in the GI tract in mice model, compared to the wild-type strain [36]. Although a detailed interaction for GbpA-N-acetylated D glucosamine was not proposed in the paper, the binding was probably mediated through hydrogen bond interactions, due to the absence of positive charges in N-acetylated D-glucosamine. Taken together, we conclude that OmpA is a direct target of CM binding, and the hydrogen bond interactions between the two molecules inhibit OmpA function, resulting in membrane disruption to cause cell death.

First, we speculated that it was unlikely that CM had positively charged free amino groups on the particle surface because i) CM were generated by cross-linking of the amino groups with sodium sulfate that would deplete free amino groups and ii) even if unoccupied amino groups remain after cross-linking, they must be deprotonated at neutral pH due to a pKa value around 6.5 [37]. However, our findings may suggest that ionic interaction is involved in the CM interaction with *E. coli* O157:H7 at neutral pH. As shown in figure 2D, the *ompA* mutant still binds to the CM-coated slides, suggesting that additional binding target(s) is involved in CM binding. One possible candidate is LPS because purified LPS is known to bind to chitosan *in vitro* [17], and we found that the *ΔwaaL* mutant, which exposes more negative charges on the cell surface, showed increased binding activity on CM-coated slides (Fig. 3D) and antimicrobial activity (Fig. 3E). In addition, antimicrobial activity increased at lower pH (pH 5) compared to the neutral pH, suggesting CM have more positive charges at acidic pH that may bind to negatively charged bacterial surface molecules, such as LPS (Fig. 3D). Therefore, we hypothesize that CM bind to *E. coli* O157:H7 via two distinct mechanisms at neutral pH. First, a hydrogen bond interaction plays a key role in OmpA-mediated binding. Second, ionic interactions contribute to the binding activity. However, positively charged CM at acidic pH bind to negatively charged surface molecules such as LPS, resulting in magnified bacterial cell death.

In order to study if CM retain antimicrobial activity *in vivo*, cows with uterine diseases were used as an animal model. Uterine disease is one of the biggest challenges facing the dairy cattle industries. Economic and revenue losses are largely impacted by these diseases as a result of the infertility, increased culling, milk production decreases, and treatment cost from uterine diseases [38], [39], [40]. Because the causes of uterine diseases are often linked to a variety of different bacteria, including IUPEC, *Trueperella pyogenes, Fusobacterium necrophorum*, and *Prevotella melaninogenica,* treatment of these diseases are often challenging with 30% failure rate [41] using conventional antibiotic treatment. As shown in figure 5, animals treated with CM showed significantly reduced IUPEC numbers in the uterine samples and was even more effective than ceftiofur treatment, suggesting that CM can be used to treat animals with these diseases. In this initial CM treatment trial, we only focused on IUPEC in the uterus, thus additional studies are needed for other pathogens, including *Trueperella pyogenes, Fusobacterium necrophorum*, and *Prevotella melaninogenica*. However, CM have a broad-spectrum of antimicrobial activity including Gram negative and positive bacteria (Fig. 4), thus we speculate that CM may have antimicrobial activity against other etiological agents of uterine disease.

For the potential application of CM, it was evaluated that CM induce prophage and overexpress Shiga-toxin in *E. coli* O157:H7 (Fig. 6). Due to the expression of Shiga-toxins during phage induction, antibiotics are not generally recommended for treatment of *E. coli* O157:H7 [42], [43]. Therefore, these results significantly emphasize the potential of CM as a therapeutic agent against this pathogen. Further studies regarding optimal CM treatment concentrations or pH effect on CM efficacy will be necessary to verify our initial findings. In addition, the broad-spectrum of antimicrobial activity of CM makes it possible to treat diseases caused by multiple pathogens such as uterine disease.

In this study, we have shown that underlying mechanisms of antimicrobial activity of CM. CM interact with OmpA protein at neutral pH, resulting in disruption of bacterial membranes and eventually leading to cell death. CM exert antimicrobial activity in an animal model with the uterine disease. Since CM treatment does not induce prophage and Shiga-toxin production, it holds promise as a treatment option for several pathogens.
